# Licorice-Induced Pseudohyperaldosteronism Highlights an Underestimated Etiology of Hypertension

**DOI:** 10.1016/j.ekir.2025.103731

**Published:** 2025-12-17

**Authors:** Jean-Joseph Bendjilali-Sabiani, Antoine Cardinale, Anaïs Lamy, Alix-Marie Pouget, Jean-Christophe Boyer, Véronique Taillard, Pascal Reboul

**Affiliations:** 1 Pharmacology and toxicology Department, Montpellier University Hospital, Montpellier, France; 2 PCCEI, Univ Montpellier, INSERM, Univ Antilles, CHU Montpellier, Montpellier France; 3 Nephrology Department, CHU Nîmes, University of Montpellier, Nîmes, France; 4 Biochemistry Department, CHU Nîmes, University of Montpellier, Nîmes, France; 5 Poison control center of Toulouse, Toulouse University Hospital, Toulouse, France; 6 Metabolic and Endocrine Diseases Department, CHU Nîmes, University of Montpellier, Nîmes, France

## Introduction

Liquorice consumption is an uncommon cause of secondary pseudohyperaldosteronism related to its glycyrrhizic acid (GZA) content. Diagnosis combines clinical presentation, biochemical abnormalities, and patient history to identify the mechanism and guide management. We report 6 cases to illustrate the toxicological impact of chronic dietary exposure to liquorice-derived GZA. These cases highlight the diagnostic value of glycyrrhetinic acid (GTA) assays in confirming glycyrrhizin intoxication and the need for increased awareness of the public health risks associated with widespread glycyrrhizin-containing products. Key teaching points of these cases are presented in Table 1.

**Table 1:** Teaching points

**Clinical** 1. Liquorice-induced pseudohyperaldosteronism should be promptly suspected in any patient presenting with hypertension, hypokalemia, and metabolic alkalosis, especially when renin and aldosterone levels are suppressed. 2. Neuromuscular symptoms such as paralysis, tetraparesis, or rhabdomyolysis may complicate severe hypokalemia and require immediate correction and supportive care.
**Biological** 3. Early determination of GTA, renin, and aldosterone concentrations is key for confirming the diagnosis and monitoring recovery after withdrawal of liquorice-containing products. 4. Hepatic dysfunction, alcohol use disorder, and interindividual metabolic variability can markedly delay GTA elimination, increasing the risk of persistent toxicity even with moderate exposure.
**Public health** 5. The widespread presence of glycyrrhizin in food, beverages, and herbal products represents a growing toxicological concern. Unintentional cumulative intake poses a public health issue that calls for stronger regulatory control and consumer education.

## Case Presentation

In Table 2, we present a summary of the collected data, including biological parameters and clinical signs. A balanced sex ratio (1:1) was observed, with a median age of 47 years. The detailed case descriptions are provided in the Supplementary Material (Case Presentation [Case 1 to 6]).

**Table 2:** A summary of the main findings of the selected cases

Case	Sex	Age, yrs	Clinical signs	Potassium level in blood (B) and urine (U)	HCO_3_^-^	Na Urine	Renin Aldosterone	Liquorice consumption	GTA	Alcohol consumption
1	M	68	HBP tetraparesis	B: 1.8 mM U: < LLOQ	46 mM	154 mM	R: < LLOQ A: < LLOQ	Alcohol-free pastis 0.5 l/d for 6 yrs	21 ng/ml 18 ng/ml	Withdrawn for 6 yrs
2	F	77	HBP	B: 2.1 mM U: 36 mM	33 mM	115 mM	A: - R: -	Antesite 2 times/d for 8 mos	49 ng/ml	Withdrawn for 6 mos
3	F	47	HBP headaches	B: 2.8 mM U: 49 mM	31 mM	< 20mM	R: 0.85 pM A: 797.5 pM	Liquorice candies 1/d since childhood	15.7 ng/ml 4.6 ng/ml	-
4	M	45	HBP tinnitus facial paralysis	B:2.4 mM U: -	29 mM	-	A: 900 pM R: -	Many liquorice candies	< 1 ng/ml	-
5	M	66	HBP bilateral finger paralysis	B: 3.3 mM U: 78 mM	28 mM	37 mM	A: - R: -	Liquorice candies	<1 ng/ml	2 glasses of wine/d
6	F	44	HBP hot flashes	B: 2.4 mM U: 32 mM	33.1 mM	61 mM	A: - R: -	Herbal tea liquorice-mint	> 100 ng/ml	3–4 glasses/d

A, aldosterone; F, female; GTA, glycyrrhetinic acid; HBP, high blood pressure; HCO_3_^-^, bicarbonates; LLOQ, low limit of quantification; M, male; R, renin.

## Discussion

GZA is the most important active ingredient in liquorice. After ingestion, it is hydrolyzed into GTA and glycyrrhetinic acid 3β-monoglucuronyl-18β-glycyrrhetinic by gut commensal bacteria (Supplementary Figure S1).^1^ Glycyrrhetinic acid 3β-monoglucuronyl-18β-glycyrrhetinic induces pseudohyperaldosteronism by dose-dependent inhibition of the 11β-hydroxysteroid dehydrogenase type 2 isoenzyme (11β-HSD2) in renal tissue.^2^ This enzyme catalyzes the conversion of cortisol and corticosterone into steroids without mineralocorticoid activity. Because cortisone has a very low affinity for mineralocorticoid receptors, 11β-HSD2 prevents excessive stimulation of mineralocorticoid receptors by cortisol. Inhibition of 11β-HSD2 is responsible for an increase in the cytosolic level of cortisol, which competes with aldosterone for binding to mineralocorticoid receptors. The inhibitory effect on 11β-HSD2 occurs even at low GTA or glycyrrhetinic acid 3β-monoglucuronyl-18β-glycyrrhetinic concentrations; the direct effect on mineralocorticoid receptors is usually negligible and is therefore only observed in cases of chronic overconsumption.^2^

In this series, the clinical and diagnostic studies as well as patient history profile were compatible with pseudohyperaldosteronism secondary to GZA intoxication. Concerning cases 1, 2, and 6, the electrocardiogram showed prolongation of the QTc interval, U or negative T waves compatible with hypokalemia. Clinically, symptoms ranged from high blood pressure, hot flashes, and headaches to tinnitus, paralysis, or tetraparesis. The biological work up showed hypokalemia in all cases, probably explaining the electrocardiogram tracing and the rhabdomyolysis described in case 1. For case 1, kaliuresis was initially low at 9 mmol/l, probably due to hypokalicystia (because of chronic intoxication). Then, the origin of hypokalemia was explored following the diagnostic pathway shown in Supplementary Figure S2, for other cases, showing high kaliuresis. Bicarbonate levels were elevated in all cases except case 5, where they were only slightly higher, likely because of the assay being performed 5 to 7 days after hospitalization. All patients tested presented with the triad of hypokalemia, hypertension, and alkalosis on initial biological work-up.

For all our cases, patient history reported consumption of liquorice and GTA assay came back positive, pointing to glycyrrhizin intoxication.^3^ A positive GTA assay supports the diagnosis of liquorice-induced pseudohyperaldosteronism by confirming glycyrrhizin intoxication in the absence of an underlying endocrine disorder. Concerning GTA levels in blood, the values ranged from residual detection (< 1 ng/ml) to > 100 ng/ml, compatible with consumption of GZA. Although most GZA concentrations observed in our cases were lower than those typically reported in the literature, clinical signs of intoxication were still present, indicating that toxicity can occur even at low exposure levels.

The highest GZA levels were observed when sampling was performed very early < 1 day after the last liquorice intake or in patients with cirrhosis (49 and 21 ng/ml in cases 1 and 2, respectively), because plasma clearance of GZA is significantly reduced because of impaired hepatic metabolism and/or biliary excretion.^4^^,^^5^ Regarding the low GTA values in cases 4 and 5, assays were performed 5 and 7 days postexposure, respectively. Given the theoretical elimination half-life of 10 to 30 hours, these findings most likely reflect residual circulating levels. A strength of our study was the accurate timing of plasma GTA measurements, allowing a more reliable interpretation of exposure dynamics. We observed the slowest elimination and higher GTA values for patients with hepatic dysfunction. Indeed, with alcohol consumption history, the calculated half-life is 128.1 hours and 27.1 hours for cases 1 and 3, respectively, which are much longer compared with the literature.^6^^,^^7^ The slower metabolism of GZA in chronic alcoholic liver disease and partial 11β-HSD2 deficiency have also been suggested.

However, GTA results must be interpreted alongside a complete endocrinological work-up to distinguish glycyrrhizin toxicity from other mineralocorticoid excess states. For case 1, endocrinological check-up showed low renin, aldosterone, and cortisol, thereby confirming liquorice intoxication. This ruled out pseudohyperaldosteronism linked to excessive secretion of mineralocorticoid hormones. Unfortunately, inconsistency in renin and aldosterone testing represents a clear limitation of the study, although diagnostic confidence was maintained through the coherent clinico-biological presentation, including markedly elevated GTA values in cases 2 and 6, and the prompt normalization of abnormalities in all cases following withdrawal of the suspected agent. Computed tomography scan and renal ultrasound were performed for all cases and revealed no adenoma, no renal artery stenosis or hyperplasia of the adrenals except for cases 3 and 4. Indeed, medical imaging showed renal artery stenosis and adrenal adenoma, respectively; thereby explaining renin and aldosterone levels. Other origins of hypermineralocorticoidism such as genetic disorders (i.e., Liddle syndrome and 11βH2 deficiency) were excluded,. Bartter and Gitelman syndrome was excluded, because the patients presented with high blood pressure.

All the patients received i.v. potassium supplementation with serial monitoring of serum potassium and blood gases until correction of metabolic disturbances. Exposure to liquorice-containing products was systematically discontinued once the diagnosis was suspected. Despite the broad and occasionally severe clinical spectrum, all patients showed progressive clinical and biochemical improvement after withdrawal and electrolyte correction. No recurrence was observed during follow-up, and none of the patients developed lasting sequelae. Each patient received targeted counselling regarding the risks associated with excessive or prolonged consumption of glycyrrhizin-containing products to prevent further episodes.

Even though all the patients were intoxicated through dietary exposure, liquorice consumption does not invariably result in severe clinical or biochemical manifestations, as illustrated by case 2. The risk of pseudohyperaldosteronism becomes significant when daily consumption exceeds 30 days, far surpassed for all cases.^8^ Toxicity depends on the dose, the type of product consumed, the mode of consumption (acute or chronic), and intra- and inter-individual variability. In case 1, the incriminating drink was alcohol-free pastis, the patient confirmed drinking 500 ml/d of this drink, for a total estimated consumption of between 175 mg and 225 mg of GZA a day. In cases 2 and 6, the consumption of Antesite or herbal licorice teas was consistent with GZA intoxication, because these products are known to have high GZA concentrations, leading to the highest blood GTA levels in our case series (Table 1). A daily intake of just 100 mg of liquorice candy was enough to increased systolic and diastolic blood pressure and lowered kaliemia.^9^ The widespread presence of glycyrrhizin in a broad range of everyday consumer products represents a significant toxicological and public health concern, because it increases the risk of inadvertent overexposure, particularly through cumulative intake from multiple sources (Supplementary Tables S1 and S2).S1–S8 This highlights the need for strengthened regulatory oversight and improved consumer awareness regarding the potential health risks associated with chronic dietary glycyrrhizin exposure. Similarly, monitoring patients undergoing alcohol withdrawal is crucial. Drinking nonalcoholic drinks as for cases 1 and 5 can be considered by a patient as a risk-free alternative to alcohol. Hypertension in a patient with alcohol use disorder, particularly in a patient consuming alcoholic pastis, should raise suspicions about a potential intake of alcohol-free beverages.

## Disclosure

All the authors declared no competing interests.

## Patient Consent

The authors declare that they have obtained consent from all the patients discussed in the report.

## Supplementary Material

Supplementary File (PDF) **Case presentation (Case 1 to 6). Supplementary References. Figure S1.** Catabolic reactions of glycyrrhizic acid in the intestine. **Figure S2.** Hypokalemia exploration. **Table S1.** Main foods and drinks containing glycyrrhizin/enoxolone. **Table S2.** Main drugs containing glycyrrhizin/enoxolone.

## Footnotes

Supplementary File (PDF)

**Case presentation (Case 1 to 6)**

**Supplementary References**
**Figure S1.** Catabolic reactions of glycyrrhizic acid in the intestine. **Figure S2.** Hypokalemia exploration. **Table S1.** Main foods and drinks containing glycyrrhizin/enoxolone. **Table S2.** Main drugs containing glycyrrhizin/enoxolone.
