# Supplementary material for: Licorice-Induced Pseudohyperaldosteronism Highlights an Underestimated Etiology of Hypertension
Source: Kidney Int Rep. 2025 Dec 17;11(3):103731. doi: 10.1016/j.ekir.2025.103731 (PMC12830285; doi:10.1016/j.ekir.2025.103731)
Supplement: Supplementary File (PDF) — Case presentation (Case 1 to 6). Supplementary References. Figure S1. Catabolic reactions of glycyrrhizic acid in the intestine. Figure S2. Hypokalemia exploration. Table S1. Main foods and drinks containing glycyrrhizin/enoxolone. Table S2. Main drugs containing glycyrrhizin/enoxolone. [file mmc1.pdf]

Supplementary material

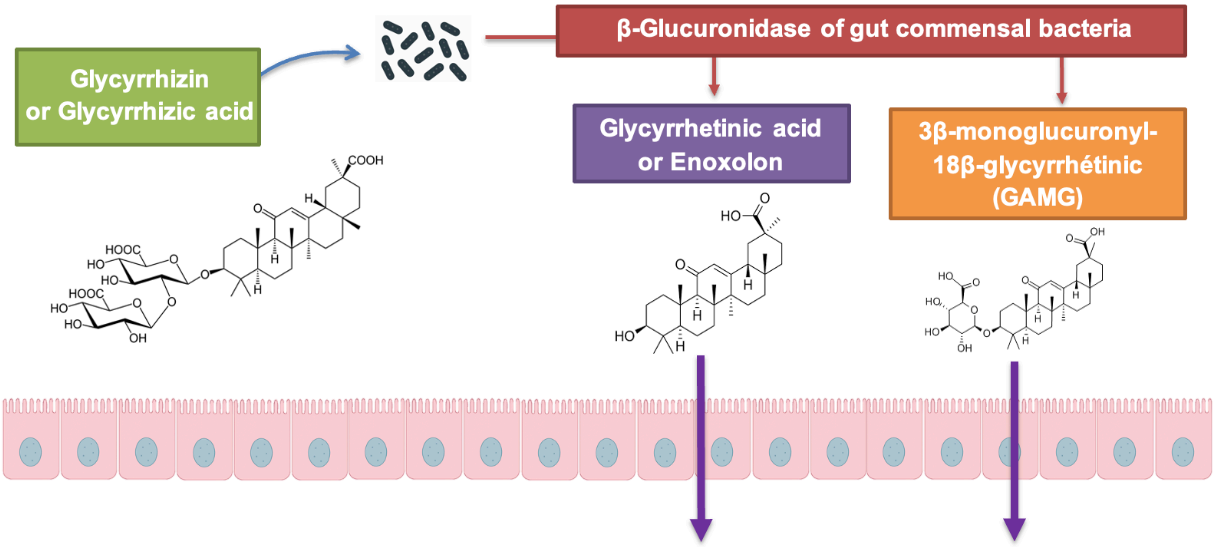

Figure S1. Catabolic reactions of Glycyrrhizic acid in the intestine.

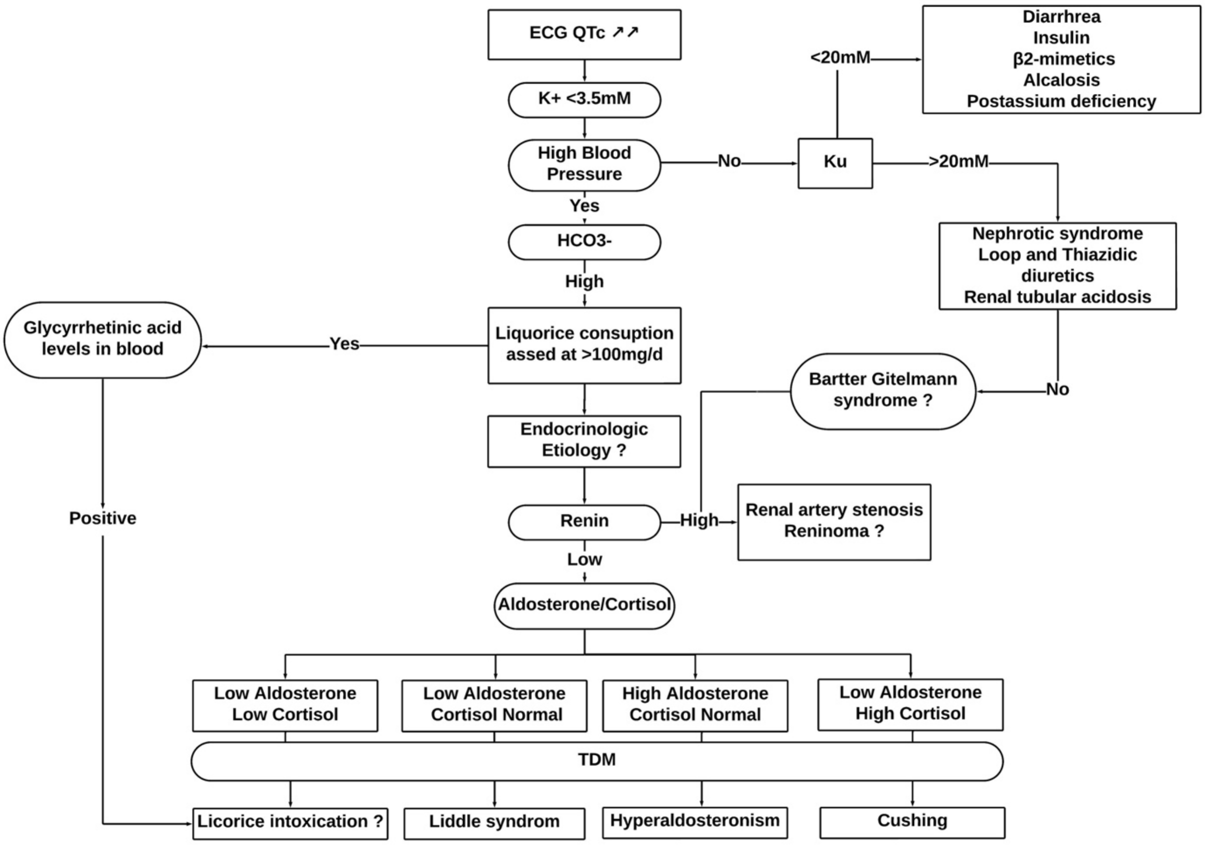

Figure S2. Hypokalaemia exploration. K^+, serum potassium; Ku, kaliuresis; HCO_3^-: serum bicarbonate.

Case presentation

Case 1

The first case was a 68-year-old man hospitalised for high blood pressure and tetraparesis. His main medical history included hypertension (treated with lercanidipin 10 mg/d), cirrhosis and chronic pancreatitis secondary to chronic alcohol use disorder assessed at 110 g of alcohol daily and weaned since August 2016. The patient mentioned long-standing bilateral burning pain in the toes, treated since June 2022. Pregabalin was given after a neurological consultation and an electromyogram showing alcoholic peripheral neuropathy. On admission, the patient had high blood pressure at 174/79 mmHg, a heart rate at 81 bpm, dysarthria and tetraparesia. The ECG showed a corrected QT interval at 471 ms, a flat T wave and a U wave. Biological tests (**Table 1**) revealed severe hypokalaemia at 1.8 mmol/L, lactates at 1.7 mmol/L, metabolic alkalosis with pH at 7.73 and very high bicarbonates at 46 mmol/L. CPK levels at 6000 IU/L suggested rhabdomyolysis. Transaminases were high with an ASAT/ALAT ratio > 2 and hypoalbuminemia reflecting a chronic alcoholic liver disease. Further questioning revealed consumption of 500mL alcohol-free pastis (AFP) a day since 2016, so a plasma GTA dosage was requested. After 8 days in hospital, the biological balance, particularly the kalaemia, was normalised via treatment with IV potassium. Clinically, the patient regained motor function in all four limbs, and his blood pressure fell to within the standards defined by the French National Authority for Health (HAS) and the international society of hypertension (ISH) at the end of his hospital stay [1]. The patient was informed of the risks of consuming this drink.

Case 2:

A 77-year-old asymptomatic woman was hospitalised due to severe hypokalaemia (2 mmol/L) and metabolic alkalosis detected during a routine blood test. She had a long-standing history of controlled hypertension since her twenties, a non-evolving adrenal nodule (discovered in 2019), type 2 diabetes since 2022, and chronic alcohol use, which she stopped 8 months prior. She also reported regular consumption of a liquorice-based syrup (Antesite™) twice daily for 8 months. On admission, her blood pressure was 150/60 mmHg, heart rate 80 bpm, and ECG showed a U wave. Biological findings confirmed hypokalaemia and elevated bicarbonates (34 mmol/L), with normal liver and pancreas function. Endocrinological evaluation showed normal urinary aldosterone, metanephrine, and normetanephrine levels, but no plasma aldosterone or renin levels were measured. Initial urinary Na/K ratio was > 1, with a Na of 115 mmol/L and K of 36 mmol/L. Upon stopping liquorice, natriuresis decreased and kaliuresis increased, alongside IV

potassium treatment. After 7 days, a plasma GTA level was found to be 49 ng/mL. The patient's blood pressure was controlled with spironolactone and perindopril after potassium normalisation. She was advised to discontinue liquorice consumption and to attend follow-up with regular medical and biological monitoring.

Case 3.

A 47-year-old woman was hospitalised for recurrent pain related to a previously diagnosed right lower pole kidney infarction confirmed by CT. Initial treatment included paracetamol/opium powder and apixaban, later switched to aspirin and analgesics. No hypertension was observed at the time. One month later, she returned with acute hypertension (180/120 mmHg), headache, and tinnitus. Repeat CT confirmed the kidney infarction, showing good re-permeabilisation of the polar artery. Blood tests revealed borderline bicarbonate and potassium levels initially, with hypokalaemia and mild metabolic alkalosis one month later. Given the hypokalaemia, alkalosis, and uncontrolled hypertension, hyperaldosteronism was suspected. Upon further inquiry, she reported long-term consumption of liquorice candy, particularly increased to 2-3 rolls per day during stress. Plasma GTA levels were 15.7 ng/mL on day 1 of hospitalisation, decreasing to 4.6 ng/mL by day 3 after stopping liquorice intake. Her endocrinology workup showed normal aldosterone and elevated renin, likely due to the kidney infarction. After discontinuing liquorice and receiving IV potassium treatment, natriuresis decreased, and kaliuresis increased. Following correction of hypokalaemia and bicarbonate normalization, hypertension was controlled with perindopril, amlodipine, and bisoprolol. She was advised to stop liquorice consumption and continue medical monitoring. Weeks later, she felt weak with hypotension, leading to a gradual reduction in antihypertensive medications.

Case 4

A 45-year-old man was hospitalised with right flank pain and no history of recent medications or toxins. He reported heavy consumption of liquorice candy in recent weeks. His blood pressure was critically high at 230/140 mmHg. Initial blood tests showed normal sodium and hypokalaemia. He was discharged with nicardipine for hypertension, oral potassium, and paracetamol. Due to persistent hypertension (220/100 mmHg), he returned to the ER with headaches, tinnitus, and paraesthesia. Repeat biological tests confirmed hypokalaemia and proteinuria (1.10 g/g). He was admitted for a hypertensive crisis with hypokalaemia. A CT scan

revealed a 15 mm benign left adrenal adenoma, confirmed by endocrinology. Renin-aldosterone testing suggested primary hyperaldosteronism, with elevated plasma aldosterone and low renin.

Doppler ultrasound ruled out renal artery stenosis. Echocardiogram showed left ventricular hypertrophy and moderate aortic dilation. Spironolactone was introduced to manage hypertension. Despite potassium supplementation, hypokalaemia persisted, prompting suspicion of resumed liquorice consumption. Plasma GTA was detected (< 1 ng/mL), supporting this theory. After treatment adjustment, his potassium normalised (4.2 mmol/L), and further monitoring for liquorice intake and hypertension management was advised.

Case 5

A 66 year-old man with a history of episodic diarrhoea, vomiting, and cramps, along with hypertension (162/94 mmHg) initially treated with perindopril and amlodipine, was admitted to the emergency room for right flank pain. He denied taking medications or toxic substances, but further questioning revealed chronic liquorice consumption in solid form. His medical history includes several hospitalisations since 2009 for uncontrolled hypertension, dizziness, and electrolyte imbalances, which were later attributed to liquorice consumption. He had episodes of severe hypertension, hypokalaemia, hypomagnesemia, hypocalcaemia, and metabolic alkalosis, linked to excessive liquorice intake, a high-salt diet, and alcohol consumption, including pastis (which also contains liquorice). Despite stopping liquorice consumption for more than six months in 2015, during which his electrolyte imbalances resolved, he was hospitalised again in 2023 for severe hypomagnesemia and hypocalcaemia. In April 2023, he was readmitted for electrolyte disturbances, including hypokalaemia, hypomagnesemia, and low calcium. Although the patient denied consuming liquorice, a plasma GTA assay was weakly positive (< 1 ng/mL), confirming chronic liquorice intoxication. Despite counselling, the patient intermittently resumed liquorice consumption, leading to recurrent hospitalisations for electrolyte imbalances. His treatment includes magnesium, potassium, calcium supplementation, and antihypertensive medications. Medical staff continue to emphasise the importance of permanently discontinuing liquorice to prevent further complications.

Case 6

A 44-year-old women was hospitalised for nutritional and endocrine evaluation for an long-standing restrictive eating disorder (body mass index of 16.3 kg/m^2, body weight 44.3 kg). She experienced unusual high blood pressure and hot flashes, treated with perindopril for one week. On admission, the patient had high blood pressure (145-150/100 mmHg) and a heart rate at 113 bpm. The ECG showed only T plate wave and a U wave. Biological tests revealed severe hypokalaemia and metabolic alkalosis (**Table 1**). The endocrinology workup showed normal chromogramin and metanephrin values. Renin and aldosterone levels were not measured. Based on the triad of hypertension, hypokalaemia and alkalosis, she was asked about any liquorice consumption. She reported consuming 3–4 mugs per day of a herbal tea containing liquorice and mint, prompting a rapid plasma GTA assay, which was highly positive (>100 ng/mL) upon admission. Serum potassium normalised after 17 days of IV potassium therapy, later switched to oral supplementation. Two months later, during a follow-up hospital visit, after discontinuing the liquorice-mint herbal tea, she was clinically well, with normal blood pressure and normal serum potassium levels, without the need for any antihypertensive medication.

	**Products**	**Glycyrrhizin levels**	**Theoretical daily quantity of food/drink for a dose of glycyrrhizin ingested > 100 mg/d**
**Drinks**	AFP	57 – 1270 mg/L	78–1754 ml<br>(3.9 – 57.7 doses of 2 cl)
	Pastis	70 – 200 mg/L	500–1429 ml<br>(25 – 71.5 doses of 2 cl)
	Liquorice Syrup	411 mg/L	243 ml<br>(12.2 doses of 2 cl)
	Antésite®	23000 mg/L	4.3 ml<br>(5.7 doses of 15 drops)
	Tea and Infusions	From<br>2 mg/L to 450 mg/L<br>(mean at 126 mg/L)	222 – 50000 ml<br>(1 to 225 cups)
**Food**	Liquorice Candies	50 to 1000 mg/kg	100 g-500 g
	Candies, Chewing-gum	0.15 to 0.2 %	-

Table S1. Main foods and drinks containing Glycyrrhizin/Enoxolone [2–4,3].

Drugs	Enoxolone levels by Intake (Tablets or Nebulisations)	Maximum dosage
**HEXALYSE®**	5 mg	8 tablets daily, equivalent to 40 mg of enoxolone
**VOCADYS®**	10 mg	8 tablets daily, equivalent to 80 mg of enoxolone
**SEDORRHOIDE®**	23 mg	3 suppositories daily, equivalent to 69 mg of enoxolone
**ANGINOVAG®**	6 mg/10 mL	3 nebulisations every 2h

Table S2. Main drugs containing Glycyrrhizin/Enoxolone [5–8].

Supplementary References

S1. Mancia G, Kreutz R, Brunström M, et al. 2023 ESH Guidelines for the management of arterial hypertension The Task Force for the management of arterial hypertension of the European Society of Hypertension: Endorsed by the International Society of Hypertension (ISH) and the European Renal Association (ERA). J Hypertens. 2023;41(12):1874-2071. doi:10.1097/HJH.0000000000003480

S2. PAYEN C, And al. Intoxication par la glycyrrhizine. VIGItox, Lyon : Centre Antipoison Centre de Pharmacovigilance, 2009 n°39.

S3. European Conmmission, Health & Consumer Protection Directorate-General, and Scientific Committee on Food. Opinion of the scientific committee on food on glycyrrhizinic acid and its ammonium salt 2003.

S4. Rosseel M, Schoors D. Chewing gum and hypokalaemia. Lancet. 1993;341(8838):175. doi:10.1016/0140-6736(93)90036-g

S5. Agencia Española de Medicamentos y Productos Sanitarios (AEMPS). Anginovag, solución para pulverización bucal. Prospecto. https://cima.aemps.es/cima/dochtml/p/37453/Prospecto_37453.html. Accessed December 22, 2023.

S6. Agence nationale de sécurité du médicament et des produits de santé (ANSM). VOCADYS, pâte à sucer. Résumé des caractéristiques du produit. https://base-donnees-publique.medicaments.gouv.fr/affichageDoc.php?specid=62459793&typedoc=R. Accessed July 13, 2023.

S7. Agence nationale de sécurité du médicament et des produits de santé (ANSM). SEDORRHOIDE CRISE HEMORROIDAIRE, crème rectale. Résumé des caractéristiques du produit. https://base-donnees-publique.medicaments.gouv.fr/affichageDoc.php?specid=66400454&typedoc=R. Accessed December 22, 2023.

S8. Agence nationale de sécurité du médicament et des produits de santé (ANSM). HEXALYSE, comprimé à sucer. Résumé des caractéristiques du produit. https://base-donnees-publique.medicaments.gouv.fr/affichageDoc.php?specid=62794329&typedoc=R. Accessed July 13, 2023.
